# Physical activity of older patients with rheumatoid arthritis

**DOI:** 10.1007/s00296-025-05901-8

**Published:** 2025-06-25

**Authors:** Yuliya Fedorchenko, Olena Zimba, Ainur B. Qumar, Marlen Yessirkepov, Burhan Fatih Kocyigit

**Affiliations:** 1 Department of Pathophysiology, Ivano-Frankivsk National Medical University, Ivano-Frankivs’k, Ukraine; 2 Department of Clinical Rheumatology and Immunology, University Hospital in Krakow, Krakow, Poland; 3 National Institute of Geriatrics, Rheumatology and Rehabilitation, Warsaw, Poland; 4 Department of Internal Medicine N2, Danylo Halytsky Lviv National Medical University, Lviv, Ukraine; 5 Department of Health Policy and Management, Asfendiyarov Kazakh National Medical University, Almaty, Kazakhstan; 6 Center for Life and Health Sciences, National Academy of Sciences Under the President of the Republic of Kazakhstan, Almaty, Kazakhstan; 7 Department of Chemical Disciplines, Biology and Biochemistry, South Kazakhstan Medical Academy, Shymkent, Kazakhstan; 8 Department of Physical Medicine and Rehabilitation, University of Health Sciences, Adana City Research and Training Hospital, Dr. Mithat Ozsan Boulevard, Kisla District, 4522 Street, No: 1, Adana, 01060 Türkiye

**Keywords:** Rheumatoid arthritis, Older adults, Physical activity, Mobility impairment, Physical function, Cerebrovascular disease, Physical Inactivity, Elderly

## Abstract

Rheumatoid arthritis (RA) in older adults presents a complex clinical challenge, exacerbated by age-related comorbidities, musculoskeletal degeneration, and psychosocial factors, all contributing to significant mobility limitations and reduced quality of life. This narrative review synthesizes current evidence on rehabilitation interventions to enhance physical function in RA patients, focusing on walking, aquatic therapy, sauna and massage therapies, and yoga. A comprehensive search of Medline/PubMed, Scopus, Web of Science, and DOAJ (up to May 2025) identified studies highlighting the efficacy of structured physical activity (PA) in reducing disease activity, fatigue, and pain, while improving functional capacity and mental health. Walking interventions, including high-intensity interval protocols, demonstrate immunomodulatory and cardiometabolic benefits, with significant reductions in Disease Activity Score (DAS28) and inflammatory markers. Aquatic therapy, leveraging water’s buoyancy, improves functional outcomes and reduces depressive symptoms, while sauna and massage therapies offer pain relief and enhanced flexibility. Yoga, as a mind–body practice, significantly lowers disease activity and enhances physical function and psychological well-being. Wearable technologies, such as actigraphy and pedometry, support personalized exercise regimens by providing real-time data for dynamic goal-setting. The European Alliance of Associations for Rheumatology (EULAR) and American College of Rheumatology (ACR) guidelines advocate for tailored exercise integration into RA management. Despite these benefits, adherence remains challenging due to pain, fatigue, and psychological barriers, necessitating individualized, biopsychosocial approaches. This review provides practical recommendations for rheumatology specialists to implement evidence-based rehabilitation strategies, emphasizing multidisciplinary care to optimize mobility and quality of life in older adults with RA.

## Introduction

**Fig. 1:**
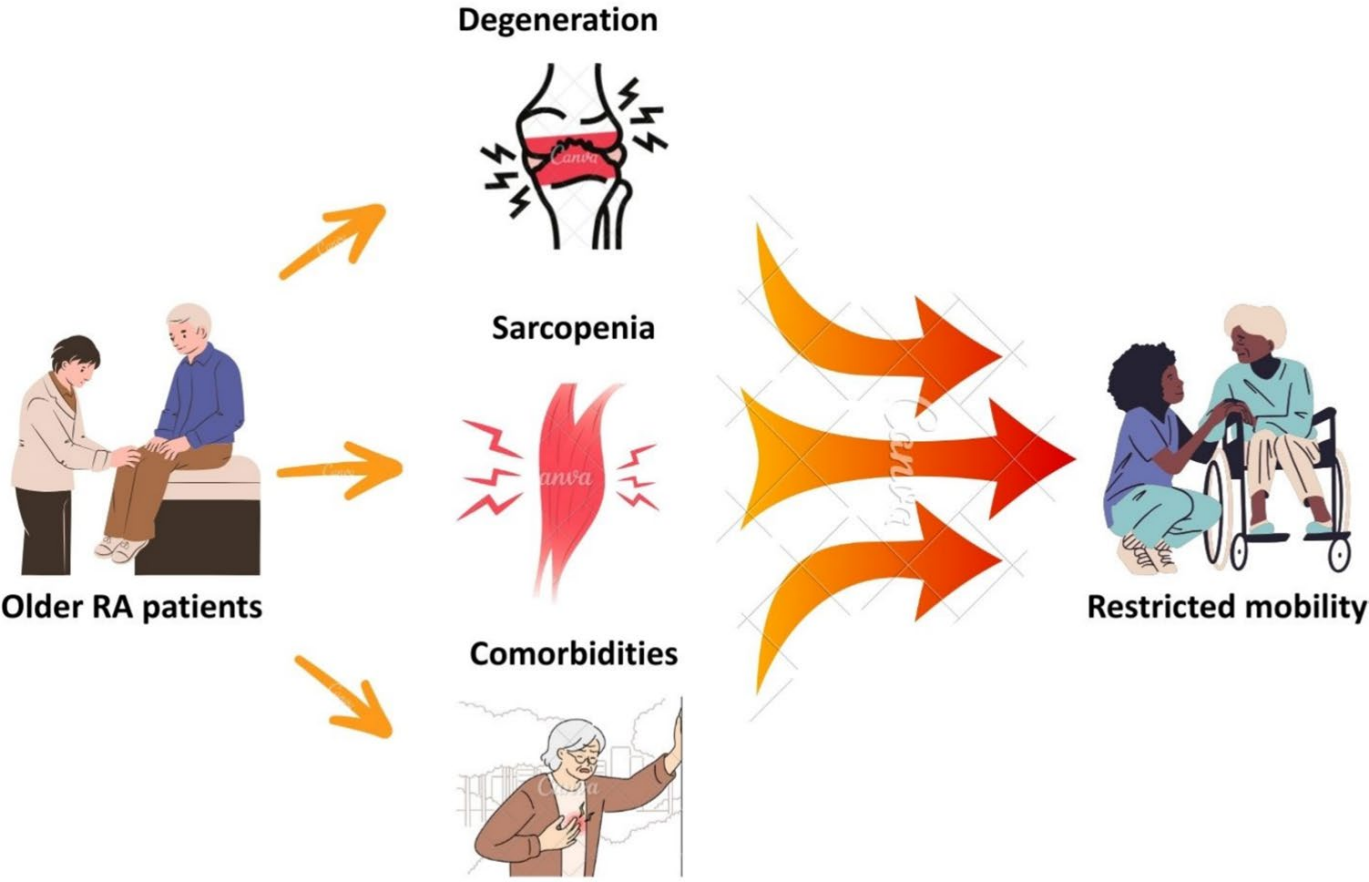
Factors contributing to restricted mobility in older rheumatoid arthritis patients

Rheumatoid arthritis (RA) is a chronic, immune-mediated inflammatory disease that primarily targets synovial joints, resulting in pain, deformity, and significant functional impairment [1]. In older adults, the clinical burden of RA is compounded by age-related musculoskeletal degeneration, sarcopenia, and the high prevalence of comorbidities, all of which restrict mobility and physical independence [2, 3] (Fig. 1). The synergistic effects of RA and aging-associated conditions lead to a profound reduction in quality of life, with mobility limitations acting as a central determinant of disability in this population.

Multiple interconnected factors contribute to impaired physical activity among elderly RA patients. Obesity, frequently observed in this group, increases mechanical stress on weight-bearing joints, amplifies systemic inflammation, and reduces exercise tolerance [4]. Coexisting osteoarthritis (OA)—especially involving the hips and knees—is common, often underdiagnosed, complicating the clinical picture and impeding effective management [5]. Moreover, cardiovascular (CVD) [6] and cerebrovascular diseases [7] are disproportionately prevalent in older RA cohorts, with exercise intolerance, fatigue, and dyspnea serving as significant barriers to physical engagement. A systematic review with meta-analysis estimated that RA confers a 1.6-fold increased risk of cardiovascular events (95% CI 1.2–2) [8]. Complementary findings from the Paracelsus 10,000 Study demonstrated a higher prevalence of carotid atherosclerotic plaques in RA patients (50%) versus non-RA controls (38%), with an adjusted odds ratio of 1.6 (95% CI 1.3–2.1), underscoring the elevated cerebrovascular risk and the utility of carotid ultrasound as a screening modality in this population [9].

Psychosocial factors also exert a considerable influence. Depression and anxiety, which are common in elderly individuals with RA, are associated with reduced motivation for physical activity, greater social isolation, and heightened disease activity [10]. Findings from a cross-sectional study revealed that, among 209 patients, the prevalence of clinically identified depression and anxiety was 10% (n = 21) and 8% (n = 17), respectively [10].

Importantly, physical inactivity in this population is not merely a passive outcome of disease; it constitutes a modifiable risk factor linked to CVD and cerebrovascular morbidity, increased frailty, and premature mortality [11]. Older RA patients often exhibit lower physical activity due to advanced joint damage, chronic pain, and fatigue, which further accelerates functional decline. Consequently, promoting physical activity should be considered a critical component of comprehensive RA management. A multinational cross-sectional study involving participants from 21 countries found that only 14% of patients exercised ≥ 3 times per week [12]. Physical inactivity was notably widespread, affecting more than 80% of participants in seven countries and 60–80% in twelve others. Inactivity correlated strongly with older age, female sex, obesity, lower educational status, comorbidities, elevated disease activity, pain, and fatigue [12].

Given these challenges, a deeper understanding of the multifactorial barriers to mobility—and the implementation of individualized, evidence-based rehabilitation strategies—is essential to improving patient outcomes. This narrative review aims to synthesize current evidence on rehabilitation interventions that may enhance mobility in older adults with RA and provide practical recommendations for rheumatology specialists, including physicians and nurses.

## Search strategy

A search was conducted across several key databases, including Medline/PubMed, Scopus, the Cochrane Library, and the Directory of Open Access Journals (DOAJ). The search methodology followed established best practices for narrative reviews [13]. The following combinations of Medical Subject Headings (MeSH) terms were used: “Rheumatoid Arthritis” AND “Physical Activity” OR “Rheumatoid Arthritis” AND “Mobility Impairment” OR “Rheumatoid Arthritis” AND “Older Adults” AND “Physical Function” OR “Rheumatoid Arthritis” AND “Cerebrovascular Disease” OR “Rheumatoid Arthritis” AND “Physical Inactivity” AND “Elderly”. Searches were limited to English-language articles published between 2020 and 2025. Only original research, systematic reviews, meta-analyses, and case reports were included. Conference abstracts, editorials, preprint articles, and book chapters were excluded from the search.

## The role of walking in modulating inflammation, psychological health, and physical function

Physical activity (PA) and sedentary behavior (SB) have emerged as modifiable lifestyle factors that significantly influence disease activity, functional capacity, and overall health outcomes in patients with RA. A growing body of evidence underscores the therapeutic value of structured PA interventions, including walking, particularly in older adults with RA, a population characterized by increased frailty, comorbid burden, and decreased physiological resilience [14].

A systematic review and meta-analysis examining lifestyle interventions targeting PA and SB revealed a modest reduction in disease activity (standardized mean difference [SMD] = –0.12; 95% CI –0.23 to –0.01; p = 0.03; I^2^ = 6%) [15]. The interventions were also associated with moderate-to-vigorous physical activity (MVPA) improvements, leisure-time activity, daily step count, fatigue, and functional ability. However, no significant effects were observed on total PA, pain intensity, anxiety, or overall health-related quality of life [15].

Another meta-analysis of randomized controlled trials assessed the impact of exercise on various health outcomes in individuals with RA [16]. The findings demonstrated that exercise significantly reduced fatigue (SMD =  − 0.28; 95% CI − 0.44, − 0.13), pain intensity (effect size [ES] =  − 0.50; 95% CI − 0.87, − 0.14), disease activity score (DAS) (weighted mean difference [WMD] =  − 0.54; 95% CI − 0.99, − 0.09; and SMD =  − 0.47; 95% CI − 0.64, − 0.30), and erythrocyte sedimentation rate (ESR) (ES =  − 0.85; 95% CI − 1.66, − 0.03). However, the exercise interventions did not significantly change grip strength, muscle strength, walk test performance, joint function, or inflammatory biomarkers [16].

High-intensity interval walking has shown promise as a safe and effective intervention in older RA patients. In a pilot study involving physically inactive individuals (mean age: 64 ± 7 years), a 10-week program of thrice-weekly, 30-min sessions led to a 9% increase in aerobic capacity (p < 0.001) alongside reductions in resting blood pressure and heart rate (p < 0.05) [17]. Critically, DAS28 decreased by 38% (p = 0.001), with accompanying declines in swollen joint counts and ESR and improvements in perceived health status [17].

On the immunological level, the intervention enhanced neutrophil function, including increased chemotaxis, phagocytosis, and reactive oxygen species (ROS) production. It also significantly reduced pro-inflammatory monocyte subsets (CD14⁺/CD16⁺) and downregulated expression of immune activation markers such as TLR2, TLR4, and HLA-DR (p < 0.05) [17]. While systemic levels of inflammatory cytokines (e.g., IL-1β, IL-6, CXCL-8, IL-10, CRP, TNF-α) remained unchanged, these cellular improvements suggest a localized immunomodulatory effect.

A survey-based longitudinal study of 345 adults with RA further supported these findings, revealing that those engaging in MVPA had lower disease activity over a three-year period [18]. However, older age (> 69 years; OR = 0.58; 95% CI 0.36–0.92), poor mental health (OR = 0.63; 95% CI 0.41–0.95), higher body mass index (BMI); (OR = 0.69–0.24; 95% CI 0.50–0.95 to 0.08–0.74), and reduced physical function (OR = 0.59; 95% CI 0.34–1.01) were key factors influencing MVPA adherence, suggesting the need for tailored interventions that address both psychological and physiological barriers [18].

Cardiometabolic benefits of PA in patients with RA have also been substantiated in randomized controlled trials. High-intensity interval training (HIIT) combined with resistance exercise significantly improved VO₂max (+ 3.71 mL/kg/min; 95% CI 2.16–5.25), oxygen pulse (+ 1.38; 95% CI 0.85–1.91), waist circumference (− 2.6 cm; 95% CI − 5.09 to − 0.18), sit-to-stand test (+ 5 reps; 95% CI 3.35–6.72), grip strength (+ 28.5; 95% CI 3.80–52.8), and overall health (− 14.7; 95% CI − 23.8 to − 5.50) though no significant effects were observed on pain, DAS28, or ESR [19].

Importantly, PA interventions—including walking and resistance training—appear to exert acute anti-inflammatory effects. A cross-sectional study demonstrated significant changes in IL-1β (p = 0.045), IL-1 receptor antagonist (IL-1ra) (p < 0.001), IL-10 (p = 0.004), IL-6 (p < 0.001), and cartilage oligomeric matrix protein (COMP) (p < 0.001), with no significant differences between groups, except for COMP, which was more sensitive in RA patients; though classical markers like CRP and TNF-α remained unchanged. These cytokine shifts suggest a transient anti-inflammatory milieu that may contribute to long-term disease modulation [20].

Furthermore, mental health status has emerged as a critical determinant of both disease trajectory and physical activity adherence [21]. A systematic review reported that PA and aerobic exercises contributed to enhanced overall well-being and a decrease in inflammation subsequently may improve cognitive function in this patient population [22].

A secondary analysis of a randomized trial found that baseline depression and anxiety predicted higher disease activity, greater joint tenderness, and reduced treatment efficacy over a two-year follow-up [23]. Given that depression and anxiety are prevalent among older adults with RA and are known to impair sexual health and intimacy, promoting walking and light PA may offer indirect benefits in these domains as well by improving mood, vitality, and body image.

Also, a survey-based study investigated the impact of PA and SB on mental health in 345 individuals with RA [24]. The results showed that light physical activity (LPA) was negatively associated with mental fatigue (β = −0.11) and depressive symptoms (β = −0.14) while positively linked to vitality (β = 0.13). Walking reduced physical fatigue (β = −0.11) and depressive symptoms (β = −0.12) and increased vitality (β = 0.15). Exercise alleviated physical (β = −0.19) and general fatigue (β = −0.12) and depressive symptoms (β = −0.09). In contrast, sedentary behavior was positively associated with physical fatigue (β = 0.19). Moderation analysis indicated that LPA improved mental fatigue and vitality in non-self-isolating individuals while walking reduced physical fatigue in self-isolating participants [24].

Finally, understanding patient motivations and barriers is essential for designing effective physical activity programs. A qualitative study identified themes such as symptom management, social support, routine integration, and communication with rheumatologists as pivotal to sustained engagement [21]. Highly active patients reported distinct perceptions of PA, emphasizing the need for individualized approaches [21].

## Swimming

Given the physical limitations and joint vulnerability commonly observed in older adults with RA, low-impact exercise modalities such as aquatic therapy have garnered increasing attention. Water’s buoyancy reduces joint stress, while resistance properties support aerobic conditioning and muscle strengthening.

The HydRA Trial, a 16-week randomized controlled intervention, investigated the comparative efficacy of water-based (WB) and land-based (LB) aerobic exercise. A total of 133 women with RA were randomized into three groups: WB exercise (n = 33), LB exercise (n = 33), and a non-intervention control group (n = 34) [25]. Primary endpoints included disease activity (DAS28), muscle strength (MS), and functional capacity assessed via the Health Assessment Questionnaire (HAQ), evaluated at baseline, 8 weeks, and 16 weeks. While no significant differences emerged in knee MS or body composition, the WB group showed statistically significant improvements in both DAS28 scores and HAQ outcomes at weeks 8 and 16 when compared to controls (p < 0.05). These findings underscore the beneficial effects of aquatic exercise in reducing disease activity and improving functional status in RA patients [25].

To further investigate the psychological impact of aquatic exercise, a quasi-experimental study evaluated its effect on pain and depressive symptoms [26]. The intervention resulted in modest physical fitness gains, with marked reductions in pain and moderate improvements in depression. Regression analysis revealed that participation significantly reduced pain (β = –20.1; 95% CI –26.2 to –14.0), which in turn was associated with a decline in depressive symptoms (β = 0.5; 95% CI 0.1–0.8). Additionally, a direct effect of the program on depression was observed (β = –14.4; 95% CI –24.1 to –4.7), with pain reduction serving as a critical mediator (indirect effect β = –9.4; 95% CI –17.7 to –2.7). These findings highlight the interconnectedness of physical and mental health in RA and position aquatic therapy as an effective adjunctive treatment for psychological comorbidities [26].

Expanding on physical outcomes, a randomized controlled trial evaluated the effects of moderate-intensity pool-based therapy on muscular endurance and vitality in RA patients [27]. Participants in the aquatic intervention group experienced significant improvements in isometric shoulder endurance, grip strength, and lower limb endurance (chair stand test), as well as vitality scores on the SF-36 (all p < 0.05), compared to the control group. Although aerobic capacity remained unchanged, the functional gains and enhanced energy levels were sustained for up to three months post-intervention. These findings suggest that aquatic exercise contributes to improved musculoskeletal performance and subjective well-being in RA, particularly in individuals for whom land-based training is challenging [27].

## Mild physical activity during massage and sauna therapies

Sauna therapy has emerged as a supportive intervention with potential benefits for RA symptom management. Regular exposure to sauna heat has been shown to attenuate joint pain, reduce stiffness, and mitigate systemic inflammation [28]. Mechanistically, thermal therapy modulates inflammatory pathways by downregulating pro-inflammatory mediators such as TNF-α, CRP, PGE2, and LTB4, while enhancing anti-inflammatory responses via IL-10. Additionally, sauna use may reduce oxidative stress and improve neuroendocrine regulation, contributing to systemic health benefits [28].

A notable case study involved a 48-year-old female RA patient who underwent a three-week multimodal therapy program comprising electroacupuncture, massage, mud therapy, and sauna sessions (EMMS) [29]. The patient demonstrated marked improvements in pain intensity, depression, anxiety, stress levels, sleep quality, and physical function post-treatment. The intervention was well tolerated, and the inclusion of mild physical activity through sauna and massage likely contributed to enhanced circulation and musculoskeletal flexibility. These results suggest the potential role of EMMS therapies as adjunctive strategies in RA management, warranting further clinical validation [29].

In support of these findings, a four-week study on infrared (IR) sauna therapy, a form of whole-body hyperthermia, showed that IR sessions were well tolerated by RA patients, with no adverse effects or disease exacerbations [30]. Participants experienced significant reductions in pain, stiffness, and fatigue immediately after treatment sessions (p < 0.05), although cumulative symptom changes did not reach statistical significance. Importantly, disease activity remained stable throughout the intervention, indicating that IR sauna therapy may offer safe, short-term symptom relief [30].

Massage therapy is another complementary approach that has demonstrated favorable effects on both the physical and psychological aspects of RA. Various massage modalities—including Swedish massage, aromatherapy massage, and foot reflexology—can reduce joint pain, improve flexibility, and modulate inflammatory and stress-related biomarkers such as IL-6, TNF-α, and cortisol [31]. In a controlled trial, Swedish massage significantly decreased pain intensity and reduced reliance on analgesics, with effects sustained up to one month after the final session (p = 0.01). These improvements were accompanied by better joint function and enhanced emotional well-being, suggesting a holistic benefit [31].

Another randomized controlled trial investigating aromatherapy massage found significant improvements in sleep disturbances and daytime fatigue across both intervention and placebo groups [32]. The aromatherapy group, however, showed a statistically significant enhancement in sleep quality during the initial weeks post-treatment (B = –1.19; 95% CI –2.35 to –0.02; p = 0.046), although no changes in pain scores were observed [32]. These findings underscore the therapeutic role of massage in improving sleep and mood, even when analgesic effects are limited.

Cold-based therapies have also attracted attention in RA management, particularly for their analgesic properties. A non-randomized clinical study compared cold air therapy and ice massage in RA patients [33]. Both methods yielded significant pain reductions immediately after treatment, with effects persisting at 30 and 60 min post-intervention (p = 0.001). No significant differences were observed between the two techniques, indicating that cryotherapy, regardless of modality, can serve as a rapid, non-invasive pain relief option [33].

## Yoga

Growing evidence supports the role of mind–body practices, particularly yoga, as effective adjunctive therapies in the management of RA. These interventions target physical and psychological domains, offering a holistic approach that may enhance the benefits of conventional pharmacologic treatment.

A systematic review and meta-analysis evaluating yoga as a complementary therapy found significant improvements in physical function, disease activity, and grip strength among RA patients [34]. Specifically, the intervention was associated with a reduction in HAQ-Disability Index scores (SMD = –0.3, 95% CI –0.6 to –0.05; I^2^ = 15%, p = 0.02) and DAS-28 scores (SMD = –0.4, 95% CI –0.7 to –0.06; I^2^ = 41%, p = 0.02), with enhanced grip strength (SMD = 1.3, 95% CI 0.5–2.1; I^2^ = 63%, p = 0.002). However, yoga did not change pain intensity, joint counts, or inflammatory biomarkers such as CRP, ESR, IL-6, and TNF-α [34].

Further support for yoga came from a systematic review and meta-analysis that included both yoga and acupuncture interventions [35]. The pooled results showed a substantial reduction in disease activity (DAS-28) across four randomized controlled trials (RCTs) (SMD = –2.5, 95% CI –2.9 to –2.1; p ≤ 0.001; I^2^ = 25.9%). Subgroup analysis isolating yoga interventions confirmed these findings, with three RCTs indicating a significant decrease in disease activity (SMD = –0.5, 95% CI –0.7 to –0.3; p ≤ 0.001; I^2^ = 0%) [35]. These outcomes highlight the growing body of evidence supporting yoga as an effective non-pharmacologic strategy for managing inflammation and disease burden in RA.

The physiological and psychological effects of yoga were further explored in a randomized controlled trial involving 72 patients with active RA. Participants underwent an 8-week yoga-based mind–body intervention alongside conventional disease-modifying antirheumatic drugs (DMARDs) therapy [36]. Compared to the control group (DMARDs only), those in the yoga group exhibited significant improvements in disease activity (DAS28-ESR, p < 0.0001; effect size = 0.210), physical function (HAQ-DI, p = 0.001; effect size = 0.159), and depressive symptoms (BDI-II, p < 0.0001; effect size = 0.5). Notably, reductions in depression were statistically associated with improvements in disease activity (R^2^ = 0.426, p < 0.0001) and functional ability (R^2^ = 0.236, p = 0.003), indicating a strong mind–body link in symptom modulation [36].

Autonomic nervous system modulation through yoga was demonstrated in a separate RCT that evaluated a 12-week yoga intervention [37]. Participants in the yoga group experienced greater reductions in disease activity compared to controls (p < 0.05), along with significantly improved autonomic function. These changes were evidenced by decreased low-frequency normalized units (LFnu), reduced LF/HF ratio, and increased high-frequency normalized units (HFnu) and total power. Furthermore, inflammatory markers and cortisol significantly reduced in the yoga group, suggesting improved sympathovagal balance and anti-inflammatory effects [37].

Although most studies report beneficial outcomes, the results depend on the primary endpoints evaluated. One RCT assessing yoga’s impact on health-related quality of life found no significant differences between intervention and control groups based on SF-36 scores (p > 0.05) [38]. Nevertheless, the yoga group showed significant reductions in fatigue (p = 0.009 at 12 weeks; p = 0.01 at 24 weeks), depression (p = 0.008 at 12 weeks), and anxiety (p = 0.025 at 24 weeks), highlighting its potential role in enhancing psychological well-being. Feasibility data indicated high adherence rates (87.5% in the yoga group vs. 82.7% in controls) and no serious adverse events, supporting the practicality and safety of implementing yoga in RA care [38].

Table 1 summarizes the main physical activity types and their documented benefits in patients with RA.

**Table 1:** Overview of physical activity types and their ımpact on rheumatoid arthritis

Physical activity type	Documented effects in RA patients
Walking (including HIIT)	↓ DAS28, ↓ pain, ↑ aerobic capacity, ↓ fatigue, improves neutrophil function, ↓ pro-inflammatory monocytes, ↑ overall well-being
Swimming	↓ DAS28, ↑ functional status, ↓ pain, ↓ depression, ↑ muscular endurance, ↑ grip strength, joint protection due to buoyancy
Massage Therapy	↓ joint pain, ↑ flexibility, ↑ emotional well-being,↑ sleep quality
Sauna Therapy	↓ joint stiffness, ↓ fatigue, improves circulation, ↑ relaxation, ↑ flexibility, ↓ oxidative stress, improves inflammatory modulation
Yoga	↓ DAS28, ↓ depression and anxiety, ↓ fatigue, ↑ grip strength, ↑ physical function, ↓ disability index, improves autonomic function

*DAS28* Disease Activity Score-28, *HIIT* High-Intensity Interval Training

## Guideline-based recommendations for physical activity

The European Alliance of Associations for Rheumatology (EULAR) and the American College of Rheumatology (ACR) underscore the vital role of exercise interventions in reducing disease activity, maintaining functional ability, and improving overall quality of life in patients with RA [39, 40].

The 2018 EULAR recommendations support incorporating aerobic, resistance, flexibility, and neuromotor exercises into the standard care of patients with inflammatory arthritis involving RA [39]. Physical activity is safe and efficient at every stage of the disease. The EULAR recommendations underline the importance of customizing exercise programs to fit individual patient profiles, considering physical limitations, comorbidities, and preferences. Regular physical activity is a collective responsibility of healthcare providers, necessitating continuous assessment and patient-centered modifications to enhance adherence and therapeutic outcomes [39].

The 2022 ACR guideline offers a comprehensive framework for integrating exercise, rehabilitation, diet, and complementary therapies with pharmacologic treatment in managing RA [40]. The ACR advocates for regular physical activity rather than inactivity, supported by moderate-certainty evidence indicating enhancements in physical function and symptom management. Specific modalities, including aerobic exercise, resistance training, aquatic therapy, and mind–body practices (such as yoga and Tai Chi), are conditionally recommended, taking into account the variability in patient tolerance, availability, and severity of the disease [40].

Current guidelines emphasize physical activity as a crucial intervention in managing RA. Implementing patient-centered exercise plans—supported by multidisciplinary teams—provides a practical approach to enhancing clinical outcomes, promoting functional independence, and improving the quality of life.

## Perspectives and strategies

Physical activity is widely recognized as a cornerstone of RA management, with robust evidence demonstrating its efficacy in mitigating joint dysfunction, enhancing muscle strength, reducing fatigue, and improving cardiovascular health [41]. Despite these benefits, adherence to physical activity guidelines remains challenging for most individuals with RA. Disease-related pain, inflammation, and persistent fatigue often impair functional mobility and reduce motivation to stay active. Consequently, developing personalized exercise regimens—tailored to disease severity, mobility limitations, and personal preferences—is essential for ensuring both feasibility and therapeutic efficacy [42].

In recent years, the integration of wearable technology into RA care has revolutionized the way clinicians monitor and evaluate physical activity. Tools such as actigraphy and pedometry offer objective, real-time data that inform both patient and clinician about physical behaviors and responses to therapy [43, 44]. These innovations have advanced the precision of treatment planning and enabled more nuanced functional status assessments.

Actigraphy, in particular, has emerged as a valuable modality for tracking continuous movement. Typically worn on the wrist or ankle, these devices measure the frequency and intensity of activity, sleep cycles, and sedentary behavior. In RA populations, actigraphy provides a longitudinal profile of physical function, capturing fluctuations in activity levels that may correspond with treatment changes, disease flares, or remissions [45]. For instance, the initiation of DMARDs or physical therapy programs can be evaluated for efficacy through alterations in step counts or movement patterns. Furthermore, actigraphy data can reinforce patient motivation by offering tangible evidence of progress, thereby supporting sustained engagement with prescribed exercise regimens [43].

While pedometers are less comprehensive, they remain a practical and accessible tool for promoting physical activity among RA patients. These step counters reliably measure daily step counts, facilitating goal-setting and adherence to low-intensity walking routines. Newer pedometers integrated with smartphone applications further enhance their utility, enabling real-time data sharing and clinician feedback [46]. This streamlined communication aids in tracking adherence, modifying activity prescriptions, and preventing overexertion, particularly during periods of heightened disease activity [46, 47].

Given the progressive nature of RA, activity recommendations should be regularly revised. Overly ambitious goals may exacerbate joint pain. Actigraphy and pedometry enable data-driven, individualized goal setting. Clinicians can emphasize low-impact activities such as gentle stretching or brief walks during disease flares. Conversely, during remissions, patients may gradually progress to moderate aerobic exercise within safe parameters [48, 49]. This adaptive approach minimizes risks and supports patient autonomy and long-term adherence to physical activity.

Beyond structured exercise, adjunctive physical therapies offer additional avenues for improving joint function and mobility. Gentle passive joint mobilizations, performed during complementary interventions like massage or sauna therapy, provide non-strenuous movement of joints within a pain-free range. When administered by trained professionals, these techniques improve synovial fluid distribution and joint lubrication [50].

Ultimately, optimal RA management necessitates a comprehensive, biopsychosocial model of care. Alongside pharmacologic therapies and physical interventions, attention to psychosocial and lifestyle dimensions is vital. Strategies that incorporate socialization, motivational psychotherapy, balanced nutrition, sleep hygiene, and routine physical activity have demonstrated beneficial effects on both disease progression and quality of life [51–53]. These integrative approaches foster sustained patient engagement, improve functional outcomes, and underscore the necessity of a multidimensional framework for managing chronic rheumatic conditions.

**Fig. 2:**
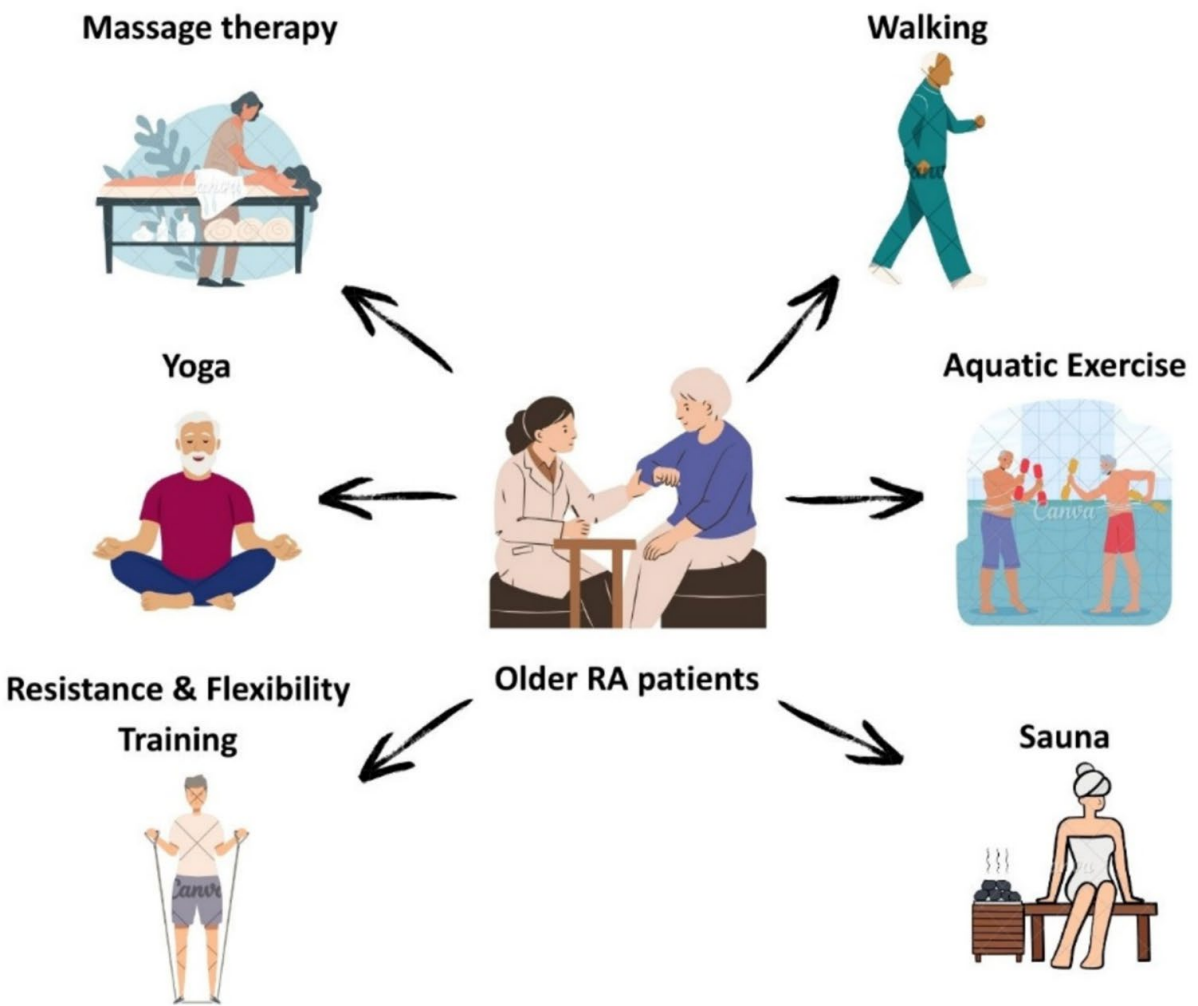
Physical activities and supportive therapies for older rheumatoid arthritis patients

Figure 2 illustrates practical examples of physical activities and supportive therapies for older patients with RA.

## Conclusions

PA and lifestyle interventions are vital in managing RA, reducing disease activity, improving function, and enhancing psychological well-being. Evidence from meta-analyses and trials shows that aerobic, resistance, aquatic, and mind–body exercises like yoga lower disease activity scores, fatigue, and pain while boosting physical and mental health. Even for older adults, HIIT and aquatic exercises are highly effective and safe.

Sedentary behavior increases fatigue and depression, while light PA, walking, and structured exercise promote resilience. Aquatic exercises reduce pain and depression, with pain relief driving mental health improvements. Complementary therapies alleviate symptoms by improving microcirculation, reducing stiffness, and upregulating anti-inflammatory cytokines like IL-10.

Wearable technologies enable personalized exercise regimens, balancing efficacy and safety. Passive joint mobilizations in therapies like massage improve mobility and synovial fluid dynamics, reducing stiffness.

A holistic approach integrating psychosocial support, nutrition, sleep, and lifestyle practices mitigates disease progression and empowers patients. These evidence-based interventions underscore the transformative potential of integrated strategies in optimizing RA outcomes.

## Data Availability

There is no stored dataset associated with the article.
